# Prediction of the need for surgery in patients with unruptured abdominal aortic aneurysm based on SOFA score

**DOI:** 10.1371/journal.pone.0314137

**Published:** 2025-01-03

**Authors:** Chao Weng, Cong Yu, Guang-wei Yang, Jin-song Jiang, Hao Wu

**Affiliations:** General Surgery, Cancer Center, Department of Vascular Surgery, Zhejiang Provincial People’s Hospital, Affiliated People’s Hospital, Hangzhou Medical College, Hangzhou, Zhejiang, China; Army Medical University, CHINA

## Abstract

**Objective:**

This retrospective study aimed to explore the association and clinical value of sequential organ failure assessment (SOFA) score on the predictors of adverse events in patients with unruptured abdominal aortic aneurysms (AAA).

**Methods:**

A total of 322 patients from Medical Information Mart for Intensive Care IV database were enrolled. Logistic regression was conducted to explore the association between SOFA and primary outcome (need for surgery, NFS). Receiver operating characteristic (ROC) and nomogram analyses were used to assess its performance for predicting NFS. We also explored the association and clinical value of SOFA on secondary outcomes including hospital length of stay (LOS), ICU-LOS, and in-hospital mortality by linear and logistic regression analyses, generalized additive model, ROC, and decision curve analysis.

**Results:**

Totally 291 patients underwent the surgery. High SOFA score significantly correlated with NFS both in crude and adjusted models (all P<0.05). SOFA had a relatively favorable prediction performance on NFS (AUC = 0.701, 95%CI: 0.596–0.802). After adjusting for related diseases, its prediction performance was increased. When SOFA was combined with lactate and gender, the model showed an AUC of 0.888 (95%CI: 0.759–1.000) and 0.3–0.9 prediction possibility. Further, the SOFA also showed significant relationship with hospital-LOS, ICU-LOS, and in-hospital mortality (all P<0.05), and exerted some value in the prediction of 7-day hospital-LOS (AUC = 0.637, 95%CI: 0.575–0.686) and in-hospital mortality (AUC = 0.637, 95%CI: 0.680–0.845).

**Conclusions:**

SOFA score was related to the NFS and can be regarded as a useful indicator for predicting the NFS in patients with AAA.

## 1 Introduction

Abdominal aortic aneurysm (AAA) is a pathologic condition with abdominal aorta diameter greater than 3 cm or dilatation exceeding the diameter of the aorta by 50% [1]. The AAA is regarded as a cardiovascular disease and its development was associated with the abnormality of smooth muscle cells originating from the splanchnic mesoderm [2]. Older age, male gender, smoking, history of hypertension, and atherosclerosis were the important risk factors of AAA. Though the AAA was more common in men, woman was likely to face a greater risk of AAA rupture [3]. Diabetes mellitus was a double-edged sword for AAA [4], and its protective effect for AAA may be due to the application of antidiabetic medications which play the anti-oxidant and anti-inflammatory effects [5]. More importantly, the rupture of AAA was related to a significantly higher rate of short-and intermediate-term death. Therefore, prevention of AAA rupture can effectively improve the prognosis of patients.

Surgical management could prevent the rupture of high-risk AAA, most commonly predicted by its size [6]. Compared with the endovascular aneurysm repair of AAA, patients who underwent the open surgical repair showed lower rates of re-intervention and stent migration, as well as better long-term outcomes [7]. A previous study has indicated that aortic wall inflammation was a useful indicator for predicting the abdominal aortic aneurysm expansion, rupture, and need for surgical repair [8]. Vascular deformation mapping has been proven to be an adjunct for surgical planning in AAA and contributed to determining the candidacy for operative repair [9]. At present, most of the studies focused on the prediction of AAA growth or rupture, the study for predicting the need for surgery in AAA was limited.

The sequential organ failure assessment (SOFA) score, an indicator for quantifying the severity of dysfunction or failure of organ systems, was commonly used to assess the risk of mortality and adverse outcomes [10]. Currently, the SOFA score has served in medical, trauma, surgical, and cardiac, and various SOFA-based models have been used to evaluate specific clinical populations [11]. In this study, we explored the potential value of the SOFA score in predicting the need for surgery in patients with AAA. Based on the SOFA score, we combined other variables and significantly increased the prediction performance of the comprehensive model.

## 2 Methods

### 2.1 Data source and study population

All the data used in this study were obtained from the Medical Information Mart for Intensive Care-IV (MIMIC-IV), a publicly available single-center critical care database. To be granted access to the database, we finished a web course named “Protecting Human Research Participants” offered by the National Institutes of Health (NIH). The approved certification number is 56208528.

In this study, the patients diagnosed with unruptured AAA admitted to ICU, aged≥18 years old, and length of hospital stay≥2 days were selected. The patients missing data of surgery records were excluded. For patients with multiple admissions records, we only retained the record on the patient’s first admission to the ICU.

### 2.2 Outcomes and covariables

In this study, the primary outcome was the need for surgery of patients with unruptured AAA, and the secondary outcomes were the hospital length of stay (LOS, days), ICU-LOS (days), and in-hospital mortality. The primary independent variable was the SOFA score. ‌SOFA score, namely sequential organ failure assessment, acts as an indicator for evaluating the degree of organ dysfunction or failure of patients and is mainly used in ICU. SOFA scoring system consists of six aspects [12], which evaluate respiratory (oxygenation index), circulation (mean arterial pressure and use of vasoactive drugs), liver (bilirubin), coagulation function (platelet), central nervous system (Glasgow coma scale score), and renal function (creatinine). The scores of each part range from 0 to 4, and the total score is calculated as SOFA score. SOFA score is usually calculated within 24 hours after the patient is admitted to ICU, and then detected every 48 hours. However, MIMIC-IV database only provided the SOFA score within 24 hours admitted to ICU. Therefore, this study only used the SOFA value within 24 hours admitted to ICU.

The demographical covariables included age (years), gender, marital status, and body mass index (BMI, kg/m^2^). The BMI was grouped into underweight (BMI<18.5 kg/m^2^), normal weight (18.5–24.9 kg/m^2^), overweight (25.0–29.9 kg/m^2^), and obese (≥30.0 kg/m^2^). The marital status was grouped into single, married, divorced, and widowed. We also collected data on histories of alcohol abuse, hypertension, renal disease, coronary heart disease (CHD), hyperlipidemia, and chronic obstructive pulmonary disease (COPD). The vital signs included heart rate (HR, bpm), respiratory rate (RR, insp/min), and blood pressure (mmHg) recorded in ICU. Only 3 patients in the non-surgery group had a record of blood pressure which limited the statistical analysis, we removed this variable in the final analysis. In addition, the laboratory blood indicators contained the anion gap (mEq/L), lactate (mmol/L), red cell distribution width (RDW, %), and glucose (mg/dL) within 24 h after admission.

### 2.3 Statistical analysis

The baseline characteristics of patients were stratified by the patient’s need for surgery (NFS). The continuous variables conforming to the normal distribution were presented as the means ± SD, and the t-test was used to compare the difference between the 2 groups. Whereas the median and quartiles were used for the nonnormal variables, and the Mann-Whitney U test was used to compare the differences. Categorical variables were expressed as frequencies and the distribution difference was analyzed by χ^2^ test.

The SOFA score was regarded as the dependent variable, and univariable linear regression analysis was performed to identify the variables correlated with the SOFA score. The mediation analysis was conducted to explore the mediating effects of potential variables on the association between SOFA score and NFS. Further, logistic regression analysis was used to explore the correlation between SOFA score and NFS. Subgroups analysis stratified by clinical features and interaction tests were performed to clarify the consistency of their correlation. The restricted cubic spline (RCS) analysis was used to visualize their relationship. In addition, receiver operating characteristic (ROC) curve analysis was used to assess the performance of SOFA score for predicting the NFS. For the prediction of the AAA surgery indication, the actual situation of the patient should also be considered, such as the patient with aging and multiple organ failure. However, the sample size in this study and positive event were limited, and it was not suitable to delete more samples based on the surgery indication. Therefore, we performed an adjusted ROC analysis to assess the prediction performance of SOFA again. We first adjusted the Charlson Comorbidity Index (CCI) in ROC analysis, which is a tool for evaluating the severity of patients’ complications and calculated by 18 indicators (such as age score, myocardial infarct, congestive heart failure, sever liver disease, metastatic solid tumor, etc.). We also adjusted several diseases variables enrolled in this study in ROC analysis. Through different adjusted ROC analyses, the prediction performance of SOFA can be comprehensively evaluated.

Then we compared the prediction performance of SOFA with other scoring systems, including Charlson Comorbidity Index (CCI) and Acute Physiology Score (APS score). APS is used to evaluate acute physiological conditions. We first used the ROC analysis and Delong test to compare the AUC difference among 3 scores. Moreover, we also performed Integrated discrimination improvement (IDI) and Net reclassification index (NRI) analyses to evaluate the degree of prediction performance improvement of SOFA relative to CCI and APS.

We next performed the multivariable backward logistic regression analysis and established an optimal comprehensive model based on 3 variables. We used the ROC and nomogram analyses to evaluate the performance of the new model for predicting the NFS. Finally, we explored the association of SOFA score with the hospital-LOS, ICU-LOS, and in-hospital mortality by linear regression and logistic regression analyses. The generalized additive model (GAM) was used to visualize the relationship between SOFA and LOS. The decision curve analysis (DCA) was performed to assess the clinical net benefit of SOFA involved in the LOS. The ROC was conducted to evaluate the prediction ability of SOFA on the 7-day LOS and in-hospital mortality. All the data were analyzed using the SPSS and R software. P<0.05 was considered statistically significant.

## 3 Results

### 3.1 Baseline characteristics of participants

In this study, a total of 322 patients with unruptured AAA admitted to the ICU were enrolled, of whom 291 patients underwent the surgery. As shown in **Table 1**, there were differences between surgery and non-surgery groups in terms of gender (P = 0.007), RDW (P = 0.022), heart rate (P = 0.049), respiratory rate (P = 0.007), anion gap (P = 0.031), and SOFA score (P<0.001). Other variables showed no differences between the two groups. Regarding 3 outcome indicators, the hospital-LOS and ICU-LOS were different between the 2 groups (all P<0.05), but in-hospital mortality showed no difference between 2 groups. We also performed logistic regression to analyze the potential variables that correlated with the SOFA score. The results (**Table 2**) showed that the distributions of hypertension (P = 0.003) and hyperlipidemia (P = 0.020) history correlated with the SOFA score. In addition, the levels of RDW (β = 0.267, 95%CI: [0.224, 0.564], P<0.001), anion gap (β = 0.306, 95%CI: [0.180, 0.390], P<0.001), lactate (β = 0.334, 95%CI: [0.401, 1.004], P<0.001), and glucose (β = 0.211, 95%CI: [0.002, 0.029], P = 0.027) were related to the SOFA score.

**Table 1:** The characteristics of the participants based on the need for surgery.

Variables	Subgroups	Non-surgery (n = 41)	Surgery (n = 291)	Statistical value	P-value
gender*	male	20	203	7.172	0.007
	female	21	88		
BMI category*	underweight	3	18	0.716	0.869
	normal weight	7	57		
	overweight	9	66		
	obese	9	91		
marital status*	single	11	51	4.665	0.198
	married	18	146		
	divorced	0	18		
	widowed	9	55		
alcohol abuse*	no	38	278	0.636	0.425
	yes	3	13		
hypertension*	no	18	149	0.766	0.381
	yes	23	142		
renal disease*	no	39	283	0.558	0.455
	yes	2	8		
CHD*	no	24	152	0.573	0.449
	yes	17	139		
hyperlipidemia*	no	19	116	0.625	0.429
	yes	22	175		
COPD*	no	25	211	2.325	0.127
	yes	16	80		
in-hospital mortality*	no	39	255	1.991	0.158
	yes	2	36		
age (years old)**		75.00±10.20	73.34±10.01	0.991	0.322
RDW (%)**		15.84±3.09	14.63±2.12	2.374	0.022
HR (bpm)***		130 [120, 130]	130 [120, 130]	-1.971	0.049
RR (insp/min) ***		18 [16, 24]	16 [14, 21]	-2.680	0.007
anion gap (mEq/L) ***		15 [13, 17]	14 [12, 16]	-2.159	0.031
lactate (mmol/L)***		1.7 [1.3, 2.9]	1.5 [1.1, 2.2]	-1.489	0.137
glucose (mg/dL)***		116.5 [102.5, 165.2]	125.5 [105.0, 160.0]	-0.320	0.749
SOFA score***		2 [1, 4]	4 [2, 6]	-3.616	<0.001
length of hospital stay***		5.131 [3.465, 7.063]	7.978 [5.090, 13.910]	-4.365	<0.001
length of ICU stay***		1.597 [1.007, 2.531]	2.307 [1.290, 4.246]	-3.018	0.003

Abbreviations: BMI, body mass index; CHD, coronary heart disease; RDW, red cell distribution width; HR, heart rate; RR, respiratory rate; SOFA, sequential organ failure assessment. The difference of variable between 2 groups was compared by χ^2^ test (*), t-test (**), and Mann-Whitney U test (***).

**Table 2:** Linear regression analysis regarding SOFA score as the dependent variable.

Variables	β		95%CI	P-value
	Unstandardized	Standardized		
gender	-0.408	-0.056	[-1.266, 0.450]	0.350
BMI category	-0.107	-0.033	[-0.504, 0.332]	0.632
marital status	-0.061	-0.019	[-0.469, 0.346]	0.768
alcohol abuse	0.947	0.060	[-0.930, 2.825]	0.321
hypertension	-1.203	-0.179	[-1.984, -0.421]	0.003
renal disease	0.757	0.040	[-1.485, 2.999]	0.507
CHD	-0.161	-0.024	[-0.955, 0.634]	0.691
hyperlipidemia	-0.949	-0.139	[-1.748, -0.149]	0.020
COPD	0.162	0.022	[-0.712, 1.036]	0.715
age	-0.002	-0.006	[-0.040, 0.037]	0.926
RDW	0.394	0.267	[0.224, 0.564]	<0.001
HR	0.000	0.001	[-0.035, 0.036]	0.987
RR	0.016	0.027	[-0.053, 0.084]	0.648
anion gap	0.285	0.306	[0.180, 0.390]	<0.001
lactate	0.702	0.334	[0.401, 1.004]	<0.001
glucose	0.015	0.211	[0.002, 0.029]	0.027

Abbreviations: BMI, body mass index; CHD, coronary heart disease; RDW, red cell distribution width; HR, heart rate; RR, respiratory rate; SOFA, sequential organ failure assessment; CI, confidence interval. The univariable linear regression analysis was performed to identify the association between these variables and SOFA score.

### 3.2 Mediation analysis for the association between SOFA score and need for surgery

We next collected the variables that correlated with the need for surgery and SOFA score, finding that RDW and anion gap were commonly related to the surgery need and SOFA score. Therefore, we speculated that the association between SOFA and the need for surgery may be mediated by RDW and anion gap. The mediation analysis (**Table** 3) showed that the direct effect of SOFA on the need for surgery was significant (Coef = 0.024, 95%CI: [0.013, 0.036], P<0.001). In addition, the anion gap (Coef = -0.004, 95%CI: [-0.009, -0.001], P<0.001) and RDW (Coef = -0.004, 95%CI: [-0.009, -0.001], P = 0.024) exerted significant mediating effects in the association between SOFA score and the need for surgery.

**Table 3:** Mediating effects of RDW and anion gap on the association between SOFA and surgery need.

Path	Coef (95%CI)	P-value
anion gap ~SOFA	0.339 [0.217, 0.462]	<0.001
RDW~SOFA	0.181 [0.103, 0.260]	<0.001
surgery~ anion gap	-0.005 [-0.016, 0.005]	0.294
surgery ~ RDW	-0.015 [-0.032, 0.001]	0.060
Total	0.016 [0.006, 0.027]	0.003
Direct	0.024 [0.013, 0.036]	<0.001
Indirect anion gap	-0.004 [-0.009, -0.001]	<0.001
Indirect RDW	-0.004 [-0.009, -0.001]	0.024

Abbreviations: SOFA, sequential organ failure assessment; RDW, red cell distribution width. The mediating effect of RDW and anion gap on the association between SOFA and surgery need was explored by the mediation analysis.

### 3.3 The association between SOFA score and the need for surgery

We further conducted the logistic regression analysis to reveal the association between SOFA score and the need for surgery. The results (**Table** 4) indicated that SOFA score was significantly related to the requirement of surgery in patients with unruptured AAA (OR = 1.362, 95%CI: [1.122, 1.652], P<0.01). After adjusting the age, gender, BMI, and marital status, a significant relationship was still observed (OR = 1.292, 95%CI: [1.031, 1.619], P<0.05).

**Table 4:** The association between SOFA and the need for surgery.

	Crude model	Adjusted model
	OR (95%CI)	OR (95%CI)
All samples		
SOFA score	1.362 [1.122, 1.652] **	1.292 [1.031,1.619] *
Subgroup analysis		
gender		
male	1.344 [1.045, 1.728] *	1.296 [0.991,1.694]
female	1.362 [1.003, 1.848] *	1.270 [0.868,1.860]
P for interaction	0.948	0.994
BMI category		
underweight	1.677 [0.432, 6.517]	0.81 [0.000, inf]
normal weight	1.390 [0.889,2.175]	1.865 [1.032,3.372] *
overweight	1.214 [0.825,1.787]	1.221 [0.817,1.823]
obese	1.214 [0.853,1.727]	1.315 [0.896,1.930]
P for interaction	0.570	0.567
hypertension		
no	1.309 [1.002,1.710] *	1.307 [0.949,1.800]
yes	1.396 [1.039,1.874] *	1.291 [0.909,1.835]
P for interaction	0.752	0.706
hyperlipidemia		
no	1.199 [0.954,1.508]	1.061 [0.798,1.411]
yes	1.603 [1.169,2.197] **	1.643 [1.123,2.402] *
P for interaction	0.145	0.384

Model 1: no variables adjusted. Model 2: adjusted the age, gender, marital status, and BMI.

*P<0.05 and

**P<0.01. Abbreviations: BMI, body mass index; SOFA, sequential organ failure assessment; OR, odds ratio; CI, confidence interval. The association between SOFA and the need for surgery was explored by logistic regression analysis and interaction effect analysis among subgroups.

We also performed the subgroup analysis to assess the sensitivity. The results revealed that the association of SOFA score with the need for surgery was not consistent. The SOFA score was shown to significantly correlate with the need for surgery in subgroups stratified by gender and hypertension history in the crude model (all P<0.05). But their relationship was not observed in the adjusted model. Interaction tests revealed that grouped variables did not significantly impact their correlation (all P for interaction>0.05).

**Fig 1:**
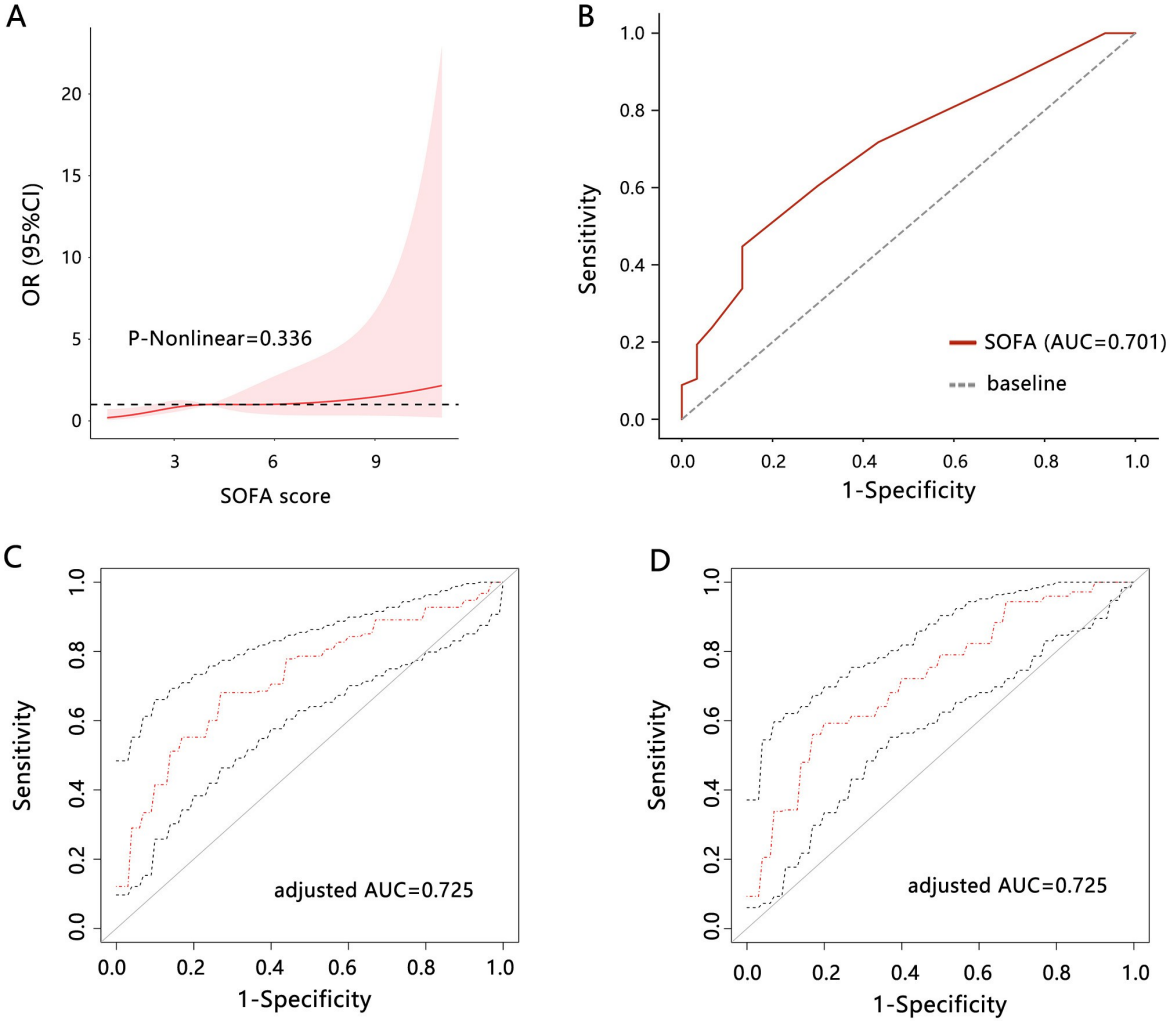
The RCS and ROC analyses. (A) RCS analysis was performed to reveal the relationship between SOFA score and the need for surgery. (B) ROC analysis was used to assess the clinical value of the SOFA score for predicting the need for surgery. (C) Adjusted ROC analysis was used to assess the clinical value of the SOFA score for predicting the need for surgery. The CCI was adjusted. (D) Adjusted ROC analysis was used to assess the clinical value of the SOFA score for predicting the need for surgery. The diabetes, hypertension, hyperlipidemia, and renal disease were adjusted. Abbreviations: SOFA, sequential organ failure assessment; RCS, restricted cubic spline; ROC, receiver operating characteristic; AUC, area under curve.

The above results have demonstrated the significant correlation between SOFA score and the need for surgery. Further, we used the RCS analysis to support their correlation (**Fig 1A**), which confirmed a linear relationship between them (P-nonlinear = 0.336).

### 3.4 Clinical value of SOFA for predicting the need for surgery

The ROC analysis (**Fig 1B**) indicated that the SOFA score had a relatively favorable performance for predicting the need for surgery in patients with unruptured AAA (AUC = 0.701, 95%CI: [0.596, 0.802]). The sensitivity and specificity were 0.448 and 0.867, respectively. The Youden’s index was 0.314 and the cutoff value of SOFA score was 5. We further adjusted the CCI calculated by 18 indicators and assessed the prediction performance of SOFA (**Fig 1C**), finding that the prediction performance of SOFA was increased (AUC = 0.725, 95%CI: [0.608, 0.801]). We also adjusted diabetes, hypertension, hyperlipidemia, and renal disease, and performed ROC analysis (**Fig 1D**), finding that the prediction performance of SOFA (AUC = 0.725, 95%CI: [0.623, 0.801]) was similar with that adjusted CCI. It followed that the adjusted prediction performance of SOFA was overmatched the unadjusted performance.

We further compared the prediction performance on need for surgery between SOFA and other 2 scoring systems including CCI and APS. The ROC analysis indicated that AUC of CCI for predicting the need for surgery was 0.524 (95%CI: [0.436, 0.641]), and AUC of APS was 0.587 (95%CI: [0.505, 0.666]). The SOFA had the largest AUC value among 3 scores, and we next compared their AUC difference. The Delong test P value between SOFA and CCI was 0.001, and it was 0.007 between SOFA and APS. In addition, we also assessed the improvement degree of SOFA prediction performance compared to CCI and APS. NRI analysis showed that SOFA had no significant improvement in predicting need for surgery than CCI and APS scores. IDI analysis indicated that prediction ability of SOFA was improved by 4.2% compared with CCI (IDI = 0.042, 95%CI: [0.012, 0.072]), and improved by 3.7% compared with APS (IDI = 0.037, 95%CI: [0.017, 0.058]). These results all highlighted the more favorable performance of SOFA than other scoring system in predicting the need for surgery in patients with unruptured AAA.

### 3.5 Model construction based on SOFA score for predicting the need for surgery

To increase the performance of the model for predicting the need for surgery, this study further introduced other valuable variables and constructed a comprehensive model. The variables separately correlated with SOFA score and the need for surgery were enrolled in the multivariable logistic regression analysis. The method of backward regression was used to establish the optimal model for predicting the need for surgery. Three variables including the gender, lactate, and SOFA score were identified in the final comprehensive model (**Table** 5). In addition, SOFA score was proved to be independently related to the need for surgery in patients with unruptured AAA (OR = 1.956, 95%CI: [1.183, 4.464], P = 0.034).

**Table 5:** Model establishment by logistic regression analysis.

	Univariable OR (95%CI)	P-value	Multivariable OR (95%CI)	P-value
gender	0.413 [0.213, 0.800]	0.009	0.085 [0.003, 0.785]	0.044
hypertension	0.746 [0.386, 1.440]	0.383		
hyperlipidemia	1.303 [0.675, 2.541]	0.430		
RDW	0.846 [0.741, 0.944]	0.004		
RR	0.937 [0.889, 0.987]	0.015		
anion gap	0.943 [0.868, 1.025]	0.169		
lactate	0.927 [0.723, 1.187]	0.547	0.505 [0.208, 0.932]	0.047
glucose	1.001 [0.983, 1.020]	0.879		
SOFA score	1.362 [1.122, 1.652]	0.002	1.956 [1.183, 4.464]	0.034

Abbreviations: SOFA, sequential organ failure assessment; RDW, red cell distribution width; RR, respiratory rate; OR, odds ratio; CI, confidence interval. The multivariable logistic regression analysis with backward method was used to establish the optimal model for predicting the need for surgery.

**Fig 2:**
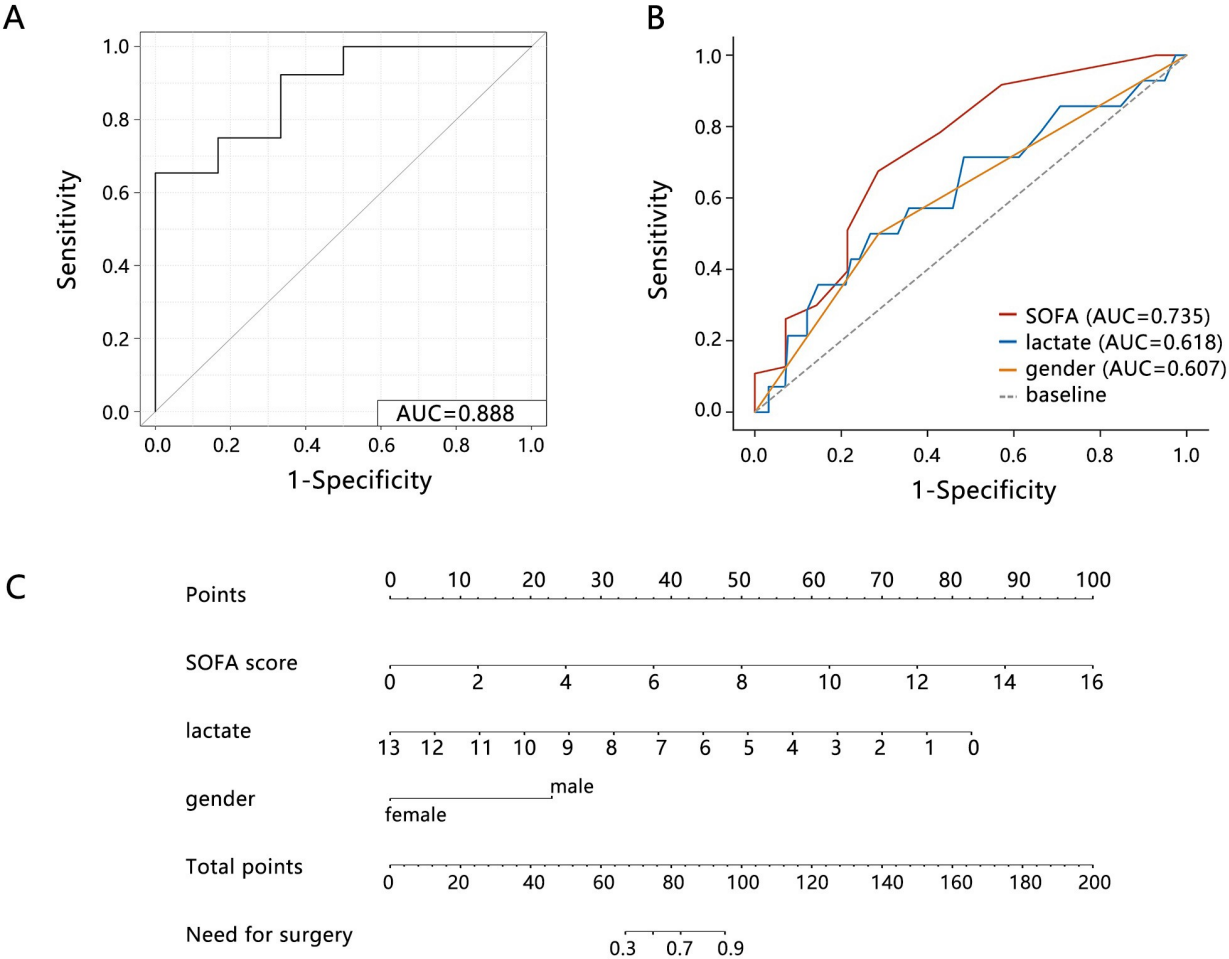
The prediction performance assessment. ROC analysis on the (A) comprehensive model based on SOFA score and (B) single variables. (C) Nomogram analysis on the comprehensive model. Abbreviations: SOFA, sequential organ failure assessment; AUC, area under curve.

The ROC analysis showed that the performance of the comprehensive model for predicting the requirement of surgery was greatly improved (**Fig 2A**, AUC = 0.888, 95%CI: [0.759, 1.000]) than that of a single SOFA score, lactate, or gender (**Fig 2B**). The nomogram analysis showed that comprehensive model can predict the 0.3–0.9 possibility for the surgery need (**Fig 2C**). These results indicated that comprehensive model based on SOFA score had potential application value in clinical.

### 3.6 Association of SOFA score with secondary outcomes

Finally, we explored the association of SOFA score with the secondary outcomes inlcuding length of hospital stay, length of ICU stay and in-hospital mortality in patients with unruptured AAA. The linear regression analysis (**Table** 6) showed that SOFA score was significantly related to the length of hoispital stay of patients both in crude (β = 0.195, 95%CI: [0.007, 0.029], P = 0.001) and adjusted models (β = 0.158, 95%CI: [0.002, 0.028], P = 0.025). Among samples that underwent the surgery, their significant association was still presented (all P<0.05). SOFA score was also significantly associated with the length of ICU stay in whole patients and those subgroup patients underwent the surgery (all P<0.01). The logistic regression analysis showed that a high SOFA score was significantly related to in-hospital mortality both in crude and adjusted models (all P<0.001).

**Table 6:** The association of SOFA score with length of stay and in-hospital mortality.

	length of hospital-stay		length of ICU-stay		in-hospital mortality	
	β (95%CI)	P-value	β (95%CI)	P-value	OR (95%CI)	P-value
All samples						
Crude	0.195 [0.007, 0.029]	0.001	0.662 [0.387, 0.936]	<0.001	1.356 [1.222–1.504]	<0.001
Adjusted model	0.158 [0.002, 0.028]	0.025	0.603 [0.258, 0.948]	0.001	1.403 [1.221–1.612]	<0.001
Samples underwent surgery						
Crude	0.270 [0.566, 1.483]	<0.001	0.673 [0.371, 0.974]	<0.001	1.371 [1.228–1.531]	<0.001
Adjusted model	0.214 [0.266, 1.325]	0.003	0.632 [0.256, 1.007]	0.001	1.429 [1.233–1.656]	<0.001

Crude: no any adjustment. Adjusted model: the age, gender, BMI, and marital status were adjusted. Abbreviations: SOFA, sequential organ failure assessment. OR, odds ratio; CI, confidence interval. The association of SOFA score with length of hospital/ICU stay was explored by linear regression analysis, and association of SOFA score with in-hospital mortality was explored by logistic regression analysis.

**Fig 3:**
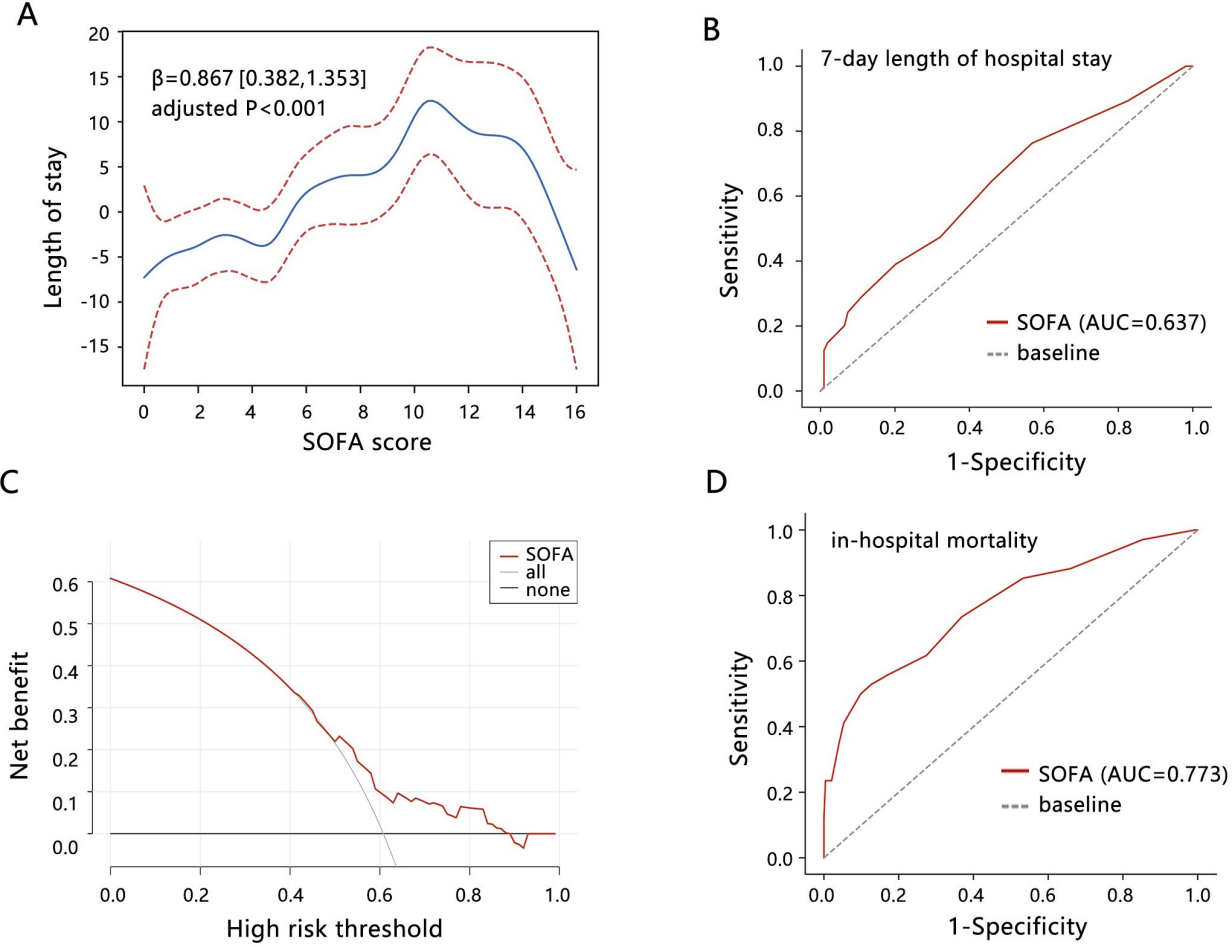
The potential value of SOFA score in terms of length of stay and in-hospital mortality in patients with unruptured AAA. (A) GAM analysis was performed to explore the relationship between SOFA score and length of stay. The age, gender, BMI, and marital status were adjusted. (B) ROC analysis was used to assess the potential of SOFA score for predicting the length of stay. (C) DCA analysis was conducted to evaluate the clinical net benefit. (D) ROC analysis was used to assess the potential of SOFA score for predicting in-hospital mortality. Abbreviations: SOFA, sequential organ failure assessment; ROC, receiver operating characteristic; AUC, area under curve; DCA, decision curve analysis.

The GAM analysis was used to further visualize their correlation, and a non-linear relationship was observed between SOFA score and length of stay (**Fig 3A**, non-linear P<0.001). Especially, a key inflection point of SOFA = 11 was observed. The length of stay increased with the SOFA score increasing when SOFA score<11. When the SOFA score>11, the length of stay decreased with the SOFA score increasing.

ROC analysis showed that the SOFA score had some value for predicting the 7-day hospital-LOS to some extent (**Fig 3B**, AUC = 0.637, 95%CI: [0.575, 0.686]). The sensitivity and specificity were 0.763 and 0.431, respectively. The DCA analysis showed that the SOFA score achieved some clinical net benefit for predicting the 7-day length of stay (**Fig 3C**). In addition, the SOFA score also showed a good performance for predicting in-hospital mortality (**Fig 3D**, AUC = 0.773, 95%CI: [0.680, 0.845]), with sensitivity, specificity, and the best cutoff value of 0.560, 0.863, and 9, respectively. These results further highlighted the prognosis value of SOFA in unruptured AAA.

## 4 Discussion

The AAA is commonly found in men and is usually asymptomatic, and the continuous dilation of aortic aneurysms can lead to the rupture which correlates with the overall 80% mortality. Approximately half of the patients with ruptured AAA who underwent intervention did not survive their hospitalization [13]. Although endovascular aneurysm repair for AAA is widely used, it is associated with increased rates of re-intervention and poor long-term outcomes [14]. Therefore, the determination of the patients needing surgery is an effective strategy to identify the high-risk populations and improve their prognosis.

The previous study showed that aortic MR elastography-derived AAA stiffness and stiffness ratio at baseline can be used to identify the potential for future aneurysm rupture or need for surgical repair [15]. At present, few studies reported a useful indicator for assessing the need for surgery (NFS). In this study, we explored the potential value of the SOFA score in predicting the need for surgery in patients with unruptured AAA. We found that a single SOFA score has achieved a relatively favorable performance for predicting the NFS. The SOFA score was commonly used as an indicator for predicting the mortality of patients [16–18]. It was also used to predict septic shock after percutaneous nephrolithotomy [19], bacteremia [20], and severity of infective endocarditis [21]. Our results indicated that SOFA is a promising predictor of NFS in patients with unruptured AAA, and its value needs further investigation.

We then found the RDW, anion gap, lactate, and glucose were related to the SOFA score. Further, anion gap and RDW exerted significant mediating effects in the association between SOFA score and the need for surgery. These results indicated the importance of anion gap and RDW on organ failure and surgery indications. RDW reflects the size of circulating erythrocytes and indicates the systemic inflammation. The production of inflammatory cytokines and corresponding inflammatory cascade have long been considered a critical pathogenic mechanism of organ failure [22]. Circulating red cells are also the mobile free radical scavengers and provide antioxidant protection to other tissues and organs. An imbalance between pro-oxidant reactions and antioxidant defense is associated with the organ failure [23]. These acknowledges may partially explain the association of RDW level with the SOFA. Previous studies also indicated the equivalent performance of RDW for the prediction of hospital mortality to SOFA score [24, 25]. The serum anion gap has been utilized to evaluate and monitor acid-base disturbances [26]. Acid-base disturbances are frequently found in ICU patients [27], and mixed acid-base disorders was related to organ failure [28]. The study found that high anion gap was associated with an increased risk of progression to kidney failure in patients with chronic kidney disease [29]. It followed that acid-base balance was critical for the maintenance of normal organ function. At present, there were no study reporting the role of anion gap and RDW on the prediction of surgery need. Related studies generally focused on their role for predicting patient’s prognosis after surgery. For example, the study suggested that RDW ≥ 13.8% was an independent predictor of postoperative infectious complications in patients with obstructive colorectal cancer [30]. Preoperative and postoperative RDW levels can preliminarily predict the effect of different metabolic surgeries in patients with obesity [31]. Elevated preoperative RDW was also associated with increased short- and long-term mortality and AKI after cardiac surgery [32]. For anion gap, it was positively correlated with the length of hospital stay in patients undergoing hip fracture surgery [33]. It was also a risk factor for predicting the severe conditions and all-cause mortality in critical ill surgical patients [34]. The potential of RDW and anion gap using in surgery indication need more investigations.

Based on the SOFA score, we significantly improved prediction performance by combining lactate and gender, of which were identified by the backward logistic regression. Previous study has shown that SOFA<9 predicted an in-hospital mortality of 16.2% and SOFA>9 predicted 73.7% mortality in ruptured AAA [35]. Although the participants enrolled in our study were the unruptured AAA, the same cutoff value of 9 for predicting the in-hospital mortality was found. In addition, our results also presented an important cutoff value of 11 as the predictor of the 7-day length of hospital stay. These researches highlighted the prognosis value of SOFA score in AAA. Regarding the lactate, it has been found that lactate level was significantly higher in the non-survivor patients with AAA [36], and preoperative arterial blood lactate levels was a predictor of hospital mortality in patients with a ruptured AAA [37]. Current studies have supported that sex plays an effect on aneurysm formation due to the differences in terms of sex hormones, the age of vasculature, and vessels size, yet are inconclusive about whether or not aneurysm formation is dependent on female/male sex hormones or a lack thereof [38]. These researches all indicated the importance of SOFA score, lactate, and gender in the development of AAA. Our study further demonstrated that the comprehensive model based on these 3 indicators showed favorable performance for predicting the need for surgery, which contributed to stratifying the high-risk populations and improved the quality of life of patients with AAA.

Finally, several limitations should be acknowledged. The sample size of participants with unruptured AAA admitted to ICU was not enough, which limited us to grouping the training and validation sets. The internal and external validations of the model’s performance were lacking in this study. In addition, the distribution of samples with the data of blood pressure and albumin in surgery and non-surgery groups did not conform with the statistical analysis, therefore these 2 variables were removed in our final analysis. The potential effects of blood pressure and albumin were ignored to some extent.

## 5 Conclusions

The SOFA score was significantly correlated with the need for surgery in patients with unruptured AAA. Even with the adjustment of related variables, their correlation was still observed. The SOFA-based model showed favorable prediction performance on the need for surgery than the single SOFA. In addition, SOFA also showed potential value in predicting the length of hospital stay.

## Supporting information

S1 Raw data (XLSX)

## References

[1] Marquez-Sanchez AC, Koltsova EK. Immune and inflammatory mechanisms of abdominal aortic aneurysm. Front Immunol. 2022;13:989933. doi: 10.3389/fimmu.2022.989933 36275758 PMC9583679

[2] Gao J, Cao H, Hu G, Wu Y, Xu Y, Cui H, et al. The mechanism and therapy of aortic aneurysms. Signal Transduct Target Ther. 2023;8(1):55. doi: 10.1038/s41392-023-01325-7 36737432 PMC9898314

[3] Boese AC, Chang L, Yin KJ, Chen YE, Lee JP, Hamblin MH. Sex differences in abdominal aortic aneurysms. Am J Physiol Heart Circ Physiol. 2018;314(6):H1137–H52. doi: 10.1152/ajpheart.00519.2017 29350999 PMC6032079

[4] Huang Z, Su H, Zhang T, Li Y. Double-edged sword of diabetes mellitus for abdominal aortic aneurysm. Front Endocrinol (Lausanne). 2022;13:1095608. doi: 10.3389/fendo.2022.1095608 36589814 PMC9800781

[5] Raffort J, Chinetti G, Lareyre F. Glucagon-Like peptide-1: A new therapeutic target to treat abdominal aortic aneurysm? Biochimie. 2018;152:149–54. doi: 10.1016/j.biochi.2018.06.026 30103898

[6] Anagnostakos J, Lal BK. Abdominal aortic aneurysms. Prog Cardiovasc Dis. 2021;65:34–43. doi: 10.1016/j.pcad.2021.03.009 33831398

[7] Siribumrungwong B, Kurita J, Ueda T, Yasui D, Takahashi KI, Sasaki T, et al. Outcomes of abdominal aortic aneurysm repairs: Endovascular vs open surgical repairs. Asian J Surg. 2022;45(1):346–52. doi: 10.1016/j.asjsur.2021.06.015 34193387

[8] Investigators MRS. Aortic Wall Inflammation Predicts Abdominal Aortic Aneurysm Expansion, Rupture, and Need for Surgical Repair. Circulation. 2017;136(9):787–97. doi: 10.1161/CIRCULATIONAHA.117.028433 28720724 PMC5571881

[9] Braet DJ, Eliason J, Ahmed Y, van Bakel PAJ, Zhong J, Bian Z, et al. Vascular Deformation Mapping of Abdominal Aortic Aneurysm. Tomography. 2021;7(2):189–201. doi: 10.3390/tomography7020017 34067962 PMC8162544

[10] Fleiss N, Polin RA. Sequential organ failure assessment scores to predict outcomes: from adults to neonates. Curr Opin Pediatr. 2023;35(2):218–22. doi: 10.1097/MOP.0000000000001207 36449658

[11] Kashyap R, Sherani KM, Dutt T, Gnanapandithan K, Sagar M, Vallabhajosyula S, et al. Current Utility of Sequential Organ Failure Assessment Score: A Literature Review and Future Directions. Open Respir Med J. 2021;15:1–6. doi: 10.2174/1874306402115010001 34249175 PMC8227444

[12] Wang XW, Niu XG, Li JX, Zhang SS, Jiao XF. SOFA Score Can Effectively Predict the Incidence of Sepsis and 30-Day Mortality in Liver Transplant Patients: A Retrospective Study. Adv Ther. 2019;36(3):645–51. 30721450 10.1007/s12325-019-0889-z

[13] De Freitas S, D’Ambrosio N, Fatima J. Infrarenal Abdominal Aortic Aneurysm. Surg Clin North Am. 2023;103(4):595–614. doi: 10.1016/j.suc.2023.05.001 37455027

[14] Seike Y, Matsuda H, Shimizu H, Ishimaru S, Hoshina K, Michihata N, et al. Nationwide Analysis of Persistent Type II Endoleak and Late Outcomes of Endovascular Abdominal Aortic Aneurysm Repair in Japan: A Propensity-Matched Analysis. Circulation. 2022;145(14):1056–66. doi: 10.1161/CIRCULATIONAHA.121.056581 35209732 PMC8969842

[15] Dong H, Raterman B, White RD, Starr J, Vaccaro P, Haurani M, et al. MR Elastography of Abdominal Aortic Aneurysms: Relationship to Aneurysm Events. Radiology. 2022;304(3):721–9. doi: 10.1148/radiol.212323 35638926 PMC9434816

[16] Schoe A, Bakhshi-Raiez F, de Keizer N, van Dissel JT, de Jonge E. Mortality prediction by SOFA score in ICU-patients after cardiac surgery; comparison with traditional prognostic-models. BMC Anesthesiol. 2020;20(1):65. doi: 10.1186/s12871-020-00975-2 32169047 PMC7068937

[17] Zhang Y, Luo H, Wang H, Zheng Z, Ooi OC. Validation of prognostic accuracy of the SOFA score, SIRS criteria, and qSOFA score for in-hospital mortality among cardiac-, thoracic-, and vascular-surgery patients admitted to a cardiothoracic intensive care unit. J Card Surg. 2020;35(1):118–27. doi: 10.1111/jocs.14331 31710762

[18] Thakur R, Naga Rohith V, Arora JK. Mean SOFA Score in Comparison With APACHE II Score in Predicting Mortality in Surgical Patients With Sepsis. Cureus. 2023;15(3):e36653. doi: 10.7759/cureus.36653 37113362 PMC10128886

[19] Shen R, Zhang W, Ming S, Li L, Peng Y, Gao X. Gender-related differences in the performance of sequential organ failure assessment (SOFA) to predict septic shock after percutaneous nephrolithotomy. Urolithiasis. 2021;49(1):65–72. doi: 10.1007/s00240-020-01190-x 32372319

[20] Ladhani HA, Sajankila N, Zosa BM, He JC, Yowler CJ, Brandt C, et al. Utility of Sequential Organ Failure Assessment score in predicting bacteremia in critically ill burn patients. Am J Surg. 2018;215(3):478–81. doi: 10.1016/j.amjsurg.2017.09.034 29089098

[21] Asai N, Shiota A, Ohashi W, Watanabe H, Shibata Y, Kato H, et al. The SOFA score could predict the severity and prognosis of infective endocarditis. J Infect Chemother. 2019;25(12):965–71. doi: 10.1016/j.jiac.2019.05.014 31320197

[22] Lv YC, Yao YH, Zhang J, Wang YJ, Lei JJ. Red cell distribution width: A predictor of the severity of hypertriglyceridemia-induced acute pancreatitis. World J Exp Med. 2023;13(5):115–22. doi: 10.5493/wjem.v13.i5.115 38173549 PMC10758662

[23] Siems WG, Sommerburg O, Grune T. Erythrocyte free radical and energy metabolism. Clin Nephrol. 2000;53(1 Suppl):S9-17. 10746800

[24] Dankl D, Rezar R, Mamandipoor B, Zhou Z, Wernly S, Wernly B, et al. Red Cell Distribution Width Is Independently Associated with Mortality in Sepsis. Med Princ Pract. 2022;31(2):187–94. doi: 10.1159/000522261 35093953 PMC9209973

[25] Lorente L, Martin MM, Argueso M, Sole-Violan J, Perez A, Marcos YRJA, et al. Association between red blood cell distribution width and mortality of COVID-19 patients. Anaesth Crit Care Pain Med. 2021;40(1):100777. doi: 10.1016/j.accpm.2020.10.013 33171297 PMC7648194

[26] Posen AK, Paloucek FP, Petzel R. Anion gap physiology and faults of the correction formula. Am J Health Syst Pharm. 2022;79(6):446–51. doi: 10.1093/ajhp/zxab423 34788391

[27] Moradi Moghaddam O, Gorjizadeh M, Sedighi M, Amanollahi A, Khatibi A, Ghodrati M, et al. Determining Predictive Power of Base Excess in Comparison with SOFA Score for Predicting Mortality in ICU Patients. Med J Islam Repub Iran. 2024;38:74. doi: 10.47176/mjiri.38.74 39399606 PMC11469720

[28] Adrogue HJ. Mixed acid-base disturbances. J Nephrol. 2006;19 Suppl 9:S97-103. 16736447

[29] Asahina Y, Sakaguchi Y, Kajimoto S, Hattori K, Doi Y, Oka T, et al. Association of Time-Updated Anion Gap With Risk of Kidney Failure in Advanced CKD: A Cohort Study. Am J Kidney Dis. 2022;79(3):374–82. doi: 10.1053/j.ajkd.2021.05.022 34280508

[30] Sato R, Oikawa M, Kakita T, Okada T, Abe T, Yazawa T, et al. Prognostic significance of the mean corpuscular volume (MCV) and red cell distribution width (RDW) in obstructive colorectal cancer patients with a stent inserted as a bridge to curative surgery. Surg Today. 2022;52(12):1699–710. doi: 10.1007/s00595-022-02504-9 35441270

[31] Zhou L, Lin S, Zhang F, Ma Y, Fu Z, Gong Y, et al. The Correlation Between RDW, MPV and Weight Indices After Metabolic Surgery in Patients with Obesity and DM/IGR: Follow-Up Observation at 12 Months. Diabetes Ther. 2020;11(10):2269–81. doi: 10.1007/s13300-020-00897-9 32789779 PMC7509025

[32] Frentiu AA, Mao K, Caruana CB, Raveendran D, Perry LA, Penny-Dimri JC, et al. The Prognostic Significance of Red Cell Distribution Width in Cardiac Surgery: A Systematic Review and Meta-Analysis. J Cardiothorac Vasc Anesth. 2023;37(3):471–9. doi: 10.1053/j.jvca.2022.11.015 36635145

[33] Yu XY, Shen JL, Xia JJ, Sun HP. The association between anion gap and length of stay in patients undergoing hip fracture surgery: data from the MIMIC-IV database. BMC Musculoskelet Disord. 2024;25(1):819. doi: 10.1186/s12891-024-07932-x 39415122 PMC11481268

[34] Sun X, Lu J, Weng W, Yan Q. Association between anion gap and all-cause mortality of critically ill surgical patients: a retrospective cohort study. BMC Surg. 2023;23(1):226. doi: 10.1186/s12893-023-02137-w 37559030 PMC10413518

[35] Krenzien F, Matia I, Wiltberger G, Hau HM, Schmelzle M, Jonas S, et al. Early prediction of survival after open surgical repair of ruptured abdominal aortic aneurysms. BMC Surg. 2014;14:92. doi: 10.1186/1471-2482-14-92 25403513 PMC4246487

[36] Song K, Guo C, Yang K, Li C, Ding N. Clinical Characteristics of Aortic Aneurysm in MIMIC-III. Heart Surg Forum. 2021;24(2):E351–E8. doi: 10.1532/hsf.3571 33798047

[37] Hashimoto M, Ito T, Kurimoto Y, Harada R, Kawaharada N, Higami T. Preoperative arterial blood lactate levels as a predictor of hospital mortality in patients with a ruptured abdominal aortic aneurysm. Surg Today. 2013;43(2):136–40. doi: 10.1007/s00595-012-0439-7 23212703

[38] Villard C, Hultgren R. Abdominal aortic aneurysm: Sex differences. Maturitas. 2018;109:63–9. doi: 10.1016/j.maturitas.2017.12.012 29452784

